# Influence of the Nd^3+^ Dopant Content in Bi_3_TeBO_9_ Powders on Their Optical Nonlinearity

**DOI:** 10.3390/ma18245545

**Published:** 2025-12-10

**Authors:** Maciej Chrunik, Alexej Bubnov, Roman Minikayev, Anastasiia Lysak, Damian Włodarczyk, Marek Nowicki, Adrian Chlanda, Marta Michalska-Domańska, Barbara Szczęśniak, Mateusz Gratzke

**Affiliations:** 1 Institute of Applied Physics, Military University of Technology, Kaliskiego 2, 00-908 Warsaw, Poland; 2 Institute of Physics, The Czech Academy of Sciences, Na Slovance 2, 182-21 Prague, Czech Republic; bubnov@fzu.cz; 3 Institute of Physics, Polish Academy of Sciences, Lotników 32, 02-668 Warsaw, Poland; minik@ifpan.edu.pl (R.M.); alysak@ifpan.edu.pl (A.L.); wlodar@ifpan.edu.pl (D.W.); 4 Faculty of Materials Engineering and Technical Physics, Poznan University of Technology, Piotrowo 3, 60-965 Poznań, Poland; marek.nowicki@put.poznan.pl; 5 Center for Advanced Technology, Adam Mickiewicz University, Uniwersytetu Poznańskiego 10, 61-614 Poznań, Poland; 6 Department of Functional Materials, Łukasiewicz Research Network—Institute of Microelectronics and Photonics, Lotników 32, 02-668 Warsaw, Poland; adrian.chlanda@imif.lukasiewicz.gov.pl; 7 Institute of Optoelectronics, Military University of Technology, Kaliskiego 2, 00-908 Warsaw, Poland; 8 Institute of Chemistry, Military University of Technology, Kaliskiego 2, 00-908 Warsaw, Poland; barbara.szczesniak@wat.edu.pl (B.S.); mateusz.gratzke@wat.edu.pl (M.G.)

**Keywords:** Bi_3_TeBO_9_, neodymium, SHG, SFD, NLO, bismuth borates, nanoparticles, rare earths, bio-medical optics

## Abstract

Second harmonic generation measurements for neodymium-doped bismuth–tellurium borate (Bi_3_TeBO_9_:Nd^3+^) powders are shown for the first time. Using undoped and low-content Nd^3+^-doped samples associated with the strongest nonlinear optical response, studies of temperature-dependent second-harmonic generation near the absorption edge were conducted. Spectroscopic measurements of the investigated powders revealed characteristic Nd^3+^ absorption bands and helped to estimate the corresponding energy band gaps for the chosen samples. The influence of low Nd^3+^-content on the absorption edge shift, as well as on the enhancement of second-harmonic generation and its temperature attenuation, is discussed. Temperature-dependent X-ray diffraction measurements enabled researchers to calculate the thermal expansion coefficients for undoped and Nd^3+^-doped Bi_3_TeBO_9_ and to assess the impact of this phenomenon on its acentricity. Thermogravimetric studies demonstrated the absence of phase transitions for the chosen samples up to their incongruent melting points. Energy Dispersive X-ray Spectroscopy measurements verified the uniformity of Nd^3+^ distribution in doped Bi_3_TeBO_9_ powders. The suitability of polycrystalline Bi_3_TeBO_9_:Nd^3+^ as media for the self-frequency doubling devices for potential optoelectronic and biomedical applications was assessed. The finest fractions of deagglomerated and suspended powders were extracted and demonstrated near-nanostructural morphology of separated particles, as revealed by means of atomic force microscopy.

## 1. Introduction

Bi_3_TeBO_9_ (BTBO) is a very promising multi-purpose nonlinear optical (NLO) crystal, reported ca. 2016 by M. Daub et al. [1] and M. Xia et al. [2]. BTBO crystallizes in polar hexagonal space group *P*6_3_, comprising a 3D framework formed by three integrated active units: stereochemically active lone-pair Bi^3+^ cations in [BiO_6_] octahedra, the Second Order Jahn–Teller (SOJT)-distorted [TeO_6_] octahedra with displaced Te^6+^, and π-orbital planar [BO_3_] groups, which enhance NLO susceptibility cooperatively [2,3,4,5,6,7]. The synergistic mechanism of these structural units was confirmed by real-space atom-cutting analysis, quantifying the contribution of each object to BTBO nonlinear coefficients [2]. Recent studies demonstrate that BTBO serves as an excellent host matrix for RE^3+^-dopants (from La^3+^ to Yb^3+^) substituting Bi^3+^ due to similarity in ionic radii, securing minimal changes of lattice parameters upon moderate doping [5,8,9,10,11,12,13,14]. Born effective charge analysis quantifies significant dynamic charge transfer in BTBO and its anisotropy, correlating with elastic and dielectric properties [3,6]. BTBO exhibits a moderate hardness and good chemical resistance [7]. Its mechanical properties show a combination of robustness and ductility (unusual in borates, many of which are brittle). Its piezoelectric response is significant, surpassing that of LiNbO_3_ and ranking second to α-BiB_3_O_6_ among borates [6,15]. Further studies revealed its strong dielectric anisotropy [3,11,16].

Undoped, polycrystalline BTBO possesses a record-high second harmonic generation (SHG) efficiency, approximately 20 times that of potassium dihydrogen phosphate (KDP), giving the largest response reported among borate NLO crystals [2]. Further research has shown that BTBO also exhibits interesting, more complex NLO properties, i.e., Photoinduced SHG (PSHG). With enhancement of the effective second-order susceptibility, BTBO induces an additional birefringence within the material tuned by an ultraviolet (UV) coherent beam near its absorption edge (Eg~3.5 eV of indirect gap) [7,16,17]. This effect demonstrates tunable photo-anisotropy and speedy optical triggering. Compared to commonly known borates (β-BaB_2_O_4_, KBe_2_BO_3_F_2_, CsLiB_6_O_10_, La_2_CaB_10_O_19_, and α-BiB_3_O_6_), BTBO is ahead of them in this category, indicating its status as a promising candidate for UV laser applications and those utilizing photoinduced anisotropic NLO effects. Contrastingly, the single-crystalline BTBO possesses noticeably weaker SHG signal compared to polycrystalline powders (0.83 vs. 20 × KDP, respectively) [1,2]. This may be due to insufficient phase-matching caused by the relatively low birefringence of BTBO (Δ*n* = 0.063) [7]. What is more, most of the isostructural RE_3_TeBO_9_ powder compounds manifest SHG signals reduced, crucially, by two orders of magnitude (~0.2 × KDP). Apart from that, BTBO melts incongruently, so the growth of its single crystals requires the flux method [1,2,6,7,10]. This route, unlike powder synthesis, leads to extremely long growth (over a few months), with the additional prospect of elevated viscosity of applied flux and therefore hindered mass flow in the melt [6,7]. Under such conditions, precise doping of BTBO crystals might be far from reliable. One possible way of increasing the SHG effect (desirable for optoelectronic and bio-medical applications) in BTBO may then prove opportune by means of introducing low-content RE^3+^ dopant into their synthesized powders, like our team reported for another NLO bismuth borate, BZBO [18,19,20,21,22,23].

For linear optics, BTBO exhibits a remarkable transparency window from near ultraviolet (NUV), across visible (VIS), to mid-infrared (MIR) (~0.35 ÷ 7.1 μm), surpassing most borate crystals’ IR cut-offs, which are below 5 μm [7]. This broad transmission is attributed to heavy atom incorporation (Bi, Te), which lowers phonon energies and suppresses multiphonon absorption, as confirmed by Raman and IR spectroscopies [3]. The BTBO absorption edge can be shifted towards NUV or VIS, depending on RE type and content [5,11]. BTBO supports a large laser damage threshold (LDT) around 450 MW/cm^2^, indicating suitability for high-power MIR laser and bio-laser applications [7,17,24]. Nd^3+^-doped BTBO exhibits NIR emissions at ~890 nm and 1064 nm, corresponding to the ^4^F_3/2_ → ^4^I_9/2_ and ^4^I_11/2_ transitions. Efficient quantum cutting processes occur via cooperative energy transfer from sensitizer Bi^3+^ ions (excited at NUV ~327 nm) to Nd^3+^ acceptors, enhancing NIR emission intensity optimally around 5 at.% Nd^3+^. Lifetime measurements revealed prolonged decay times (~108 μs for 0.5 at.% doping), which decreased with higher Nd^3+^ concentrations, due to luminescence concentration quenching (LCQ) facilitated by multipolar interactions [5,9]. Co-doping BTBO with Yb^3+^ and Er^3+^, as confirmed by excitation spectra and decay kinetics, makes it promising for spectral up-conversion luminescence, recently desirable in photovoltaic converters and biomedical optics [12,25,26]. Bulk ceramics of BTBO:Yb^3+^/Er^3+^ show an increase of emission lifetimes (up to 175 µs), depending on the pressure applied during sintering [27].

BTBO’s powders offer a unique integration of the features described above and provide a model for the deliberate structural design of next-generation NLO materials. By combining strong SHG, broad optical transparency, high dielectric response, mechanical ductility, and doping ability, BTBO stands out among borates and other NLO crystals. With its multifunctionality and a balanced combination of mechanical and optical properties, BTBO is positioned as a versatile “all-in-one” medium. Relevant applications include tunable solid-state lasers, optical switches, up-conversion devices, radiation detectors, multi-band frequency converters, and bio-optical sensors, pointing to BTBO:RE^3+^ as a promising material for optoelectronics. This illustrates how targeted structural integration can push the limits of NLO material performance across multiple application domains. Compared to other borates, BTBO combines sets of desired and promising features, whereas the other materials typically compromise on one attribute for the sake of another [5,16,17]. Optimized doping balances luminescence yield with retention of the host’s exceptional NLO performance, creating multifunctional matrices for advanced photonics and bio-optics [9,11,12,13,19,20,21,22,23,24,25,26]. Co-doping of BTBO can additionally facilitate cross-relaxation or energy-transfer up-conversion, improving both lasing and SHG [5,28].

Self-frequency doubling (SFD) is an NLO process in which a RE^3+^-doped crystal simultaneously generates laser emission and doubles its fundamental frequency [5,28,29,30,31]. The first SFD device was developed in 1969 in co-doped LiNbO_3_:Tm^3+^/Nd^3+^, and the technique was also demonstrated in RE^3+^-doped borates such as YAB and GAB [30,31,32,33]. SFD materials must meet stringent requirements: (1) ability to perform RE-doping with excellent lasing capability, broad transparency combined with numerous absorption bands, long fluorescence lifetime, high quantum efficiency, and low phonon energy; (2) high NLO coefficients and birefringence for phase matching; (3) good thermal conductivity and stability, low thermal expansion anisotropy; and (4) physical robustness, chemical resistance, and non-hygroscopic nature [34,35]. The scarcity of SFD materials—especially among borate crystals—arises from the conflict in the need for both high-symmetry structures (ideal for lasing) and the absence of inversion symmetry (required for SHG), as well as limitations in doping capability for many borates (e.g., α-BiB_3_O_6_, LiB_3_O_5_, and KBe_2_BO_3_F_2_) [34]. A significant part of the above-mentioned requirements can be met by Nd^3+^-doped BTBO. Neodymium ions play a pivotal role in SFD crystals due to the strong luminescent properties, narrow emission bands, and long fluorescence lifetimes of these crystals, resulting in lasing possibilities and gaining efficiency in nonlinear conversion [36,37,38,39]. In our previous studies, we confirmed some of the features mentioned [9]. In this work, we expand current research with results and findings on BTBO:Nd^3+^ powders to assess their potential applications as materials for SFD devices.

## 2. Materials and Methods

### 2.1. Synthesis of BTBO:Nd^3+^ Samples

Polycrystalline powder samples of undoped and Nd^3+^-doped BTBO were synthesized by means of the modified Pechini method [40]; a detailed description is in our previous work [9]. The following compounds were obtained: Bi_3_TeBO_9_, Bi_2.985_Nd_0.015_TeBO_9_, Bi_2.97_Nd_0.03_TeBO_9_, Bi_2.925_Nd_0.075_TeBO_9_, and Bi_2.85_Nd_0.15_TeBO_9_ (hereafter simply labeled as BTBO, BTBO:Nd^3+^(0.5%), BTBO:Nd^3+^(1.0%), BTBO:Nd^3+^(2.5%) and BTBO:Nd^3+^(5.0%), respectively). Additionally, a set of BTBO:RE^3+^(1.0%) with some other lanthanides (RE = Tm, Ho, Pr, and Er) was prepared using the same procedure to allow for comparative analysis in current and future work.

### 2.2. Samples Characterization

#### 2.2.1. SHG Measurements at Room Temperature

**Figure 1:**
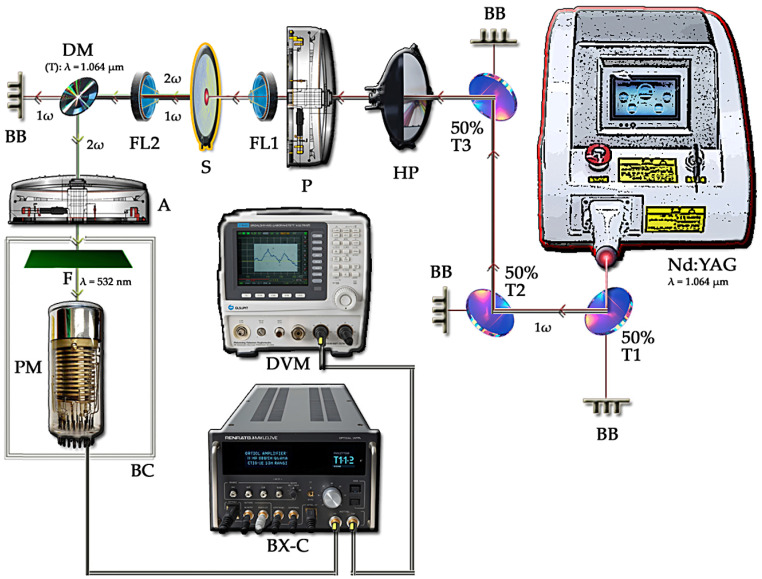
Scheme of opto-electronic setup used for SHG measurements at RT (where Nd:YAG—source laser, 50% T(1,2,3)—semi-reflective mirrors, HP—half-wave plate, P—polarizer, FL1, FL2—focusing lenses, S—sample, DM—dichroic mirror, reflective for SHG, A—analyzer, F—optical filter, PM—photomultiplier, BB—beam blocker, BC—box cover, BX-C—boxcar-type amplifier, and DVM—digital voltmeter).

The SHG study of the investigated samples at room temperature (RT) was performed using the Kurtz–Perry (KP) method designed for powder polycrystalline samples [18,41,42]. The experimental setup is shown in Figure 1. As a first harmonic source, we used a Q-switched Nd:YAG laser (~0.2 mJ/pulse, λ = 1.064 µm, 6-ns pulses with repetition equal to f = 20 Hz and ca. 4 mm spot diameter). As a reference for the quantitative measure of SHG efficiency, we used a KDP (potassium dihydrogen phosphate, KH_2_PO_4_) polycrystalline sample [43]. Pulses which were quite weak (0.2 mJ) were used to prevent the local overheating of samples, to avoid additional intrinsic effects. The experiment was performed on fine-powder samples, which were pressed into thin layers with a thickness not exceeding 100 µm and placed between two microscopic glass plates. The research was conducted using a mode that was purely transmission.

#### 2.2.2. SHG Measurements at Higher Temperatures

**Figure 2:**
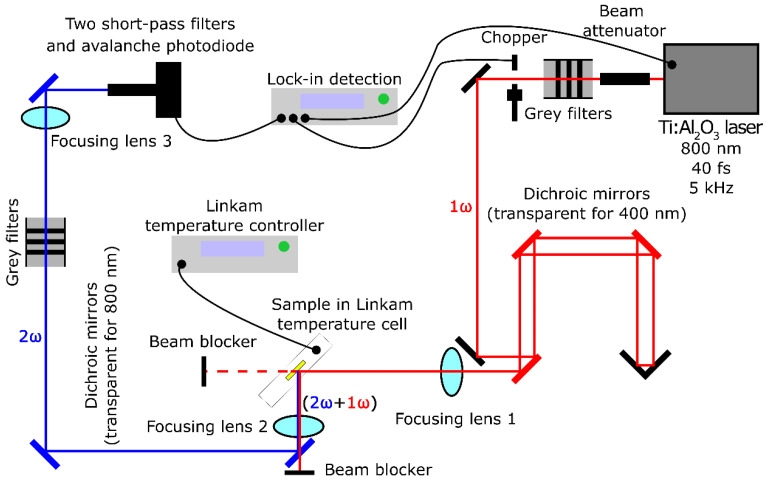
Scheme of the opto-electronic setup used for SHG measurements at different temperatures.

For the temperature-dependent SHG investigations, a setup similar to the opto-electronic setup referred to in Kremer et al. [44] was used. The Ti:Al_2_O_3_ femtosecond laser amplifier Spitfire ACE (Spectra-Physics, Santa Clara, CA, USA) was used, producing 40 fs long pulses with a first harmonic of λ = 800 nm and a pulse repetition rate of f = 5 kHz. The radiant exposure of individual pulses was set to ∼0.01 mJ/cm^2^. The collimated first harmonic laser beam reached the sample, which was placed into a Linkam heating stage equipped with a temperature controller providing an accuracy of ±0.1 K. Thorlabs 0.2 mm powder-filled cuvettes were secured for the measurements. These cuvettes were positioned at 45° to the direction of the incident beam (due to the adopted reflection-measurement mode). The SHG signal generated in the sample was subsequently spectrally filtered with optical dichroic mirrors of a transmissive first harmonic wavelength of λ = 800 nm (remaining reflective for SHG). The signal output was detected with an avalanche photodiode and subsequently amplified with a Lock-in amplifier. No standardized samples were used for these measurements. The described setup is depicted in detail in Figure 2**.**

#### 2.2.3. UV-VIS Spectroscopic Studies

The spectrometric analysis was performed using a Lambda 650 UV-Vis spectrometer (Perkin Elmer) fitted with an integrating sphere. Diffusive reflectance spectra (DRS) were gathered within the 200 to 800 nm wavelength interval at RT. The optical band gap (BG) energies for both doped and selected undoped BTBO:Nd^3+^ powders were derived from the spectrometry data, following the method described by Haryński et al. [45]. This technique involves expanding the logarithmic form of the Tauc equation by using the Taylor series, and is suitable for both transmission and reflection modes. The Tauc exponent is extracted from the slope of the tangent to the absorption data. Understanding this coefficient provides insight into the nature of optical transitions and serves as an input for BG calculations. Additionally, in the previously employed method, it was sometimes noted that a part of the graph perpendicular to the x-axis did not converge to zero, affecting BG determination and resulting in a significant underestimation of it [46]. Application of the method outlined by Haryński et al. [45] eliminates this issue.

#### 2.2.4. XRD Investigations

All the XRD diffraction patterns of as-synthesized: undoped and Nd^3+^-doped BTBO powders, as recorded at RT, as well as their corresponding crystallographic calculations and results, have previously been reported in our previous papers [3,9]. These mentioned studies were supplemented this time, for selected samples, with temperature-dependent powder diffraction measurements made using a PANALYTICAL X’pert Pro MPD diffractometer (PANALYTICAL, Malvern, UK), equipped with CuK_α_ radiator, collecting data in the operative mode at 40 kV voltage and 25 mA current (no monochromator) and Bragg–Brentano geometry. Each measurement was performed with at least a 0.015° step size, and also involved a semiconducting strip-detector. An Anton Paar HTK-1200 N temperature chamber was applied to stabilize the temperature in the range from RT to 220 °C. Rietveld refinement and precise calculations of the complete diffraction profiles, along with refined unit cell parameters, were performed using the FullProf Suite (Ver. January-2021) [47]. The crystallite sizes of as-prepared powders were estimated by means of the Debye−Scherrer method [48].

#### 2.2.5. DTA/TG Measurements

Differential thermal analysis and thermogravimetric (DTA/TG) measurements for undoped and selected Nd^3+^-doped BTBO samples were carried out using a NETZSCH STA 449 F5 (NETZSCH, Selb, Germany) apparatus in uncovered Al_2_O_3_ crucibles (0.3 mL in volume) under synthetic air flow (50 mL/min, 80% N_2_/20% O_2_) at constant heating rate (5 K/min, same for cooling) in a 30–920 °C temperature range. Each sample weight was ca. 50.0 ± 0.1 mg.

#### 2.2.6. EDX Studies

The records of powder morphology for undoped and selected Nd^3+^-doped samples were obtained using a scanning electron microscope SEM/FEI Quanta 250FEG (FEI Company, Eindhoven, The Netherlands). The samples were analyzed in the Low-Vacuum Mode at a pressure of 70 Pa. The images were recorded in secondary electron mode at an accelerating voltage of 5 kV. For microchemical analysis, the EDX spectra were performed with an EDAX Octane SDD system at an accelerating voltage of 30 kV. The mentioned set of results of the microelemental quantitative analysis was previously reported in our previous work in raw form [9]. In the present work, we are focusing on considerations of the Nd^3+^/Bi^3+^ ratio as the most reliable indicator of dopant content instead of individual elements.

For EDX mapping of studied samples, a Hitachi SU-70 scanning electron microscope coupled with a Thermo Fisher Scientific (Waltham, MA, USA) Energy Dispersion X-ray spectrometer equipped with a Li-drift silicon X-ray detector and the Norah System 7 was used. The elemental distribution EDX maps were collected under an accelerating voltage of 20 kV to ensure the penetration of the entire powder particle depth.

#### 2.2.7. AFM Sample Preparation and Topography

The synthesized undoped and selected Nd^3+^-doped powders of BTBO were deagglomerated by means of a physical method using ultrasonication. Each powder sample was dispersed in chloroform (CHCl_3_) and placed in a 25 cm^3^ beaker. No additional surfactants or particle stabilizers were used. The suspension was put in the VTUSC3 (VELLEMAN, Gavere, Belgium) ultrasonic washer, working at 170 W with an ultrasound frequency of 42 kHz. The process was carried out for at least 1 h using deionized water as a refrigerant; then, the beaker contents were poured into a chemical cylinder and left for the self-sedimentation process, which required several hours. After that, about 1 cm^3^ was taken from the top of such suspension with the use of a glass pipette. In the next step, about a dozen suspension drops, containing the finest powder fraction, were placed on a microscopy glass slide and spread over the substrate. It was then left for 24 h at RT, allowing the liquid phase to evaporate from the dispersive phase.

Finally, all three obtained specimens (BTBO, BTBO:Nd^3+^(0.5%), and BTBO:Nd^3+^(1.0%)) were subjected to AFM examination, using the Dimension FastScan (BRUKER, Billerica, MA, USA). The aforementioned samples, deposited on microscope slides, were directly installed on the microscope’s table. The measurement system of the microscope consisted of an Icon scanning head, which was equipped with an ACT scanning probe (App Nano, Mountain View, CA, USA). To protect the system from acoustic artifacts, the microscope was enclosed in a chamber provided by the manufacturer. Before the study, the drive frequency of the scanning probe was determined to be approximately 281 kHz. Other probe parameters, such as the tip radius (below 10 nm) and the spring constant (around 37 N/m), were set according to the manufacturer’s data. The study was conducted at RT (approximately 22 °C) in an air atmosphere, and by using the Tapping Mode. This allowed for the acquisition of high-resolution 2D and 3D images of the examined powders. After the study, the images were analyzed, and the average particle size was determined using the freeware software Jens Rüdigs Makroaufmaßprogrammm ver. 0.9.2.

## 3. Results and Discussion

### 3.1. Effect of Nd^3+^ Content on Nonlinear Optical Behavior of BTBO:Nd^3+^

**Figure 3:**
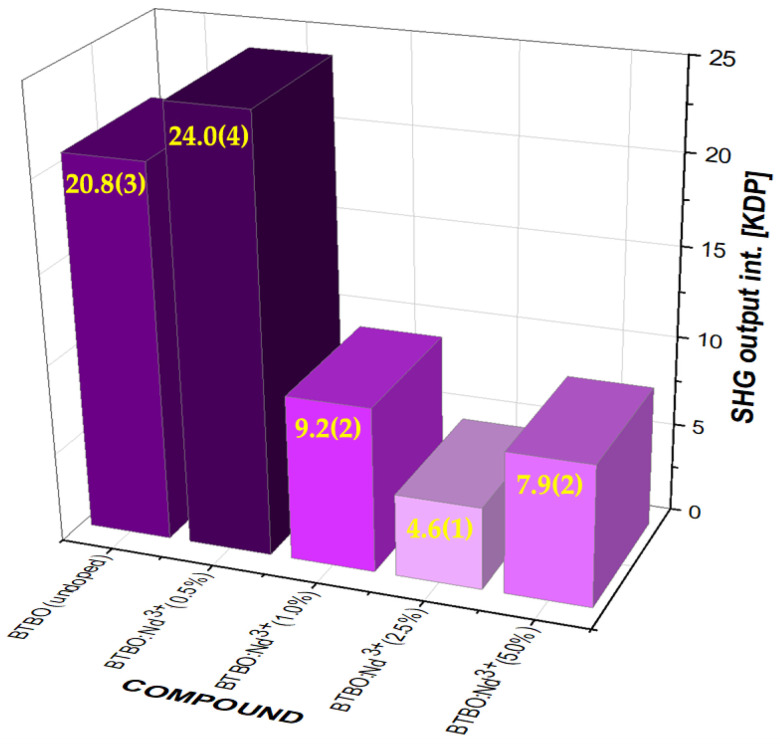
A comparison between the SHG intensities measured for undoped and Nd^3+^-doped BTBO powder samples (with reference to KDP SHG taken as 1.0) with different dopant concentrations. The numbers in parentheses are the standard uncertainties of the measurement.

The applied measurement method (KP) made it possible to conduct reliable qualitative and quantitative research. Two series of measurements were performed to determine the influence of the Nd^3+^ dopant content in the BTBO matrix on its second-order nonlinear optical response (SHG). The first series included the complete set of synthesized materials (from an undoped one to 5 at.% Nd^3+^) and was conducted exclusively at RT. The results of these measurements are included in Figure 3.

In the KP method, each of the tested samples was no thicker than 0.1 mm. The relationship between the laser beam spot diameter (*d_B_*), sample thickness (*L*), and the average powder particle size ($\hat{r}$) is as follows:

(1) $$d_{B}\gg L\gg \hat{r},$$

and when the sample is transmissive both for 1ω and 2ω, the radial distribution of the SHG signal for the backward and forward beams is close to cosine (and fulfills Lambert’s law [49]). The sample then behaves as an isotropic, planar radiator operating in a quasi-transmissive mode. When these criteria are met, the intensity of the second-harmonic electric field component $E_{i}^{2\omega }(L)$ can be described as follows:

(2) $$E_{i}^{2\omega }\left(L\right)=-i\omega \varepsilon_{0}\sqrt{\frac{\mu_{0}}{\varepsilon_{2i}}}\sum_{j,k}d_{ijk}E_{j}^{\omega }E_{k}^{\omega }\left(\frac{e^{i\Delta k_{ijk}L}-1}{i\Delta k_{ijk}}\right),$$

where *i*, *j*, *k*—directions consistent with the polarization eigenvectors, *µ*_0_—magnetic permeability of vacuum, *ɛ*_2*i*_—electric permittivity of the material for the $E_{i}^{2\omega }(L)$ field component from SHG, Δ*k_ijk_*—the difference in the wave vectors of the excited and absorbed photons, and *d_ijk_*—the optical nonlinearity coefficient of the material [50]. Under these standardized conditions, the KP method is reliable and repeatable.

It can be seen that undoped BTBO showed an SHG signal with good agreement with the values already reported by Xia et al. [2] (only 0.8 × KDP unit higher than previously). This slightly stronger signal could have been caused by a different synthesis method (solid-state reaction vs. the Pechini method used here), a factor which most likely contributed to obtaining powder particles with nano- and micrometric fractions (as we prove later, in Section 3.5 and Section 3.6), the more developed surface imperfections and internal defects of which caused an increase in the number of scattering centers and, consequently, additionally enhanced the second harmonic signal. In turn, the effect of substituting Bi^3+^ ions with Nd^3+^ was the most crucial even at the lowest dopant concentration (0.5 at.%), for which the strongest optical nonlinearity signal was observed (enhancement by over 15% compared to undoped BTBO). Further doping leads to a threshold reduction in SHG of more than half (observed for 1.0 at.% Nd^3+^), and then the signal weakens even more, but without significant fluctuations (for 2.5 and 5.0 at.%). Such a threshold weakening in the signal, preceded by its distinct enhancement, is a very unusual phenomenon for such a range of dopant concentrations. In the previous work, we found that for BTBO:Nd^3+^(0.5%), we had the longest luminescence lifetime (emission at λ = 1.06 μm), and by increasing the dopant concentration, this was successively shortened [9]. However, we do not believe that it is related to something like concentration quenching (for example, R. Cong et al. [51] previously demonstrated for δ-BIBO:Nd^3+^ that luminescence quenching exists but does not affect SHG at all, which can be constant even up to 15 at.% of neodymium dopant). In turn, in another work by our group, we demonstrated that a similar effect (relative to these findings) occurred for BZBO:Tb^3+^ and also in a narrow dopant range [18]. All of the above suggests that much depends, both qualitatively and quantitatively, on the dopant, and largely on the matrix itself. It can also be assumed that this is because SHG emission occurs near the characteristic absorption band for neodymium (described in detail later in Section 3.2). As it turns out, these bands can actually be wider and deeper for higher concentrations of this particular dopant, while for 0.5 at.% they are still very negligible, but at the same time, such a small neodymium content improves disproportionately the polarization of the medium and its nonlinear susceptibility (possibly by means of resonant enhancement for 4f-4f transitions at this concentration—especially when the dopant could occupy the internodal positions). It should be noted, however, that the SHG signal at the level of 4–8 × KDP still represents a measurable and non-trivial nonlinear optical response. To illustrate this, Table 1 compares the best results from this section of the current work with those reported in the past for other noncentrosymmetric borate crystals.

Based on the results shown in Figure 3, it can be suggested that the greatest SHG enhancement, in the case of BTBO doping with neodymium ions, should be sought in the Nd^3+^ concentration range from 0.0 to 1.0 at.%. It seems quite likely that in these narrow concentration ranges (slightly smaller or slightly larger than 0.5 at.%), an even more efficient frequency doubling could be observed. Already at this stage, it can be concluded that the acentricity of the BTBO matrix is very sensitive to small changes in its base stoichiometry, and in certain ranges this leads to an improvement of this noncentrosymmetry. Although the vast majority of SFD material systems involve single crystals operating in the transmission mode, the current study demonstrates that the use of a measurement system modeled as the one for the KP method—with some modifications—would allow, to some extent, the use of thin films of powder materials to observe the SFD effect in the quasi-transmission mode.

**Figure 4:**
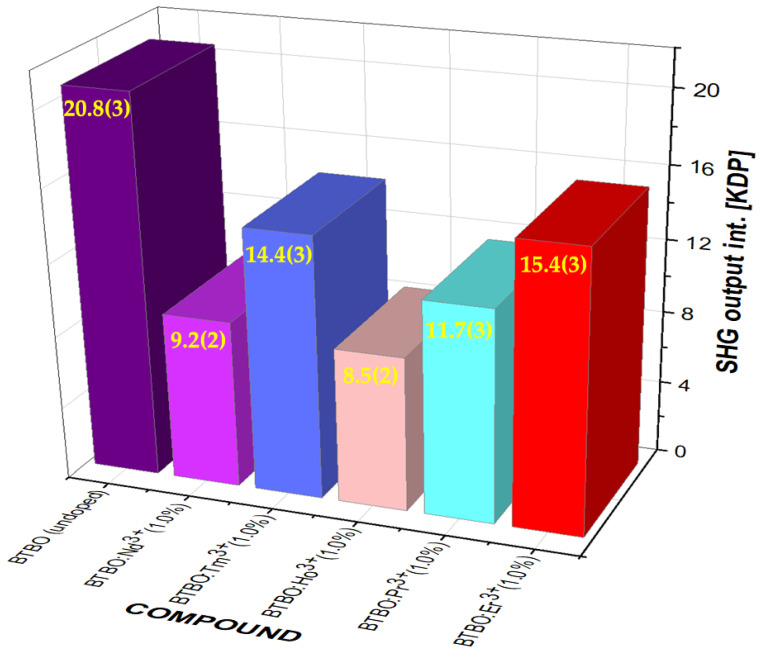
A comparison between the SHG intensities measured for undoped and various RE^3+^-doped (each at 1.0 at.%) BTBO powder samples (with reference to KDP SHG taken as 1.0). The numbers in parentheses are the standard uncertainties of the measurement.

To complement and extend this stage of research, four other BTBO:RE^3+^ (RE = Tm, Ho, Pr, and Er) variants were examined, each with the same concentration of lanthanide ions (1 at.%) and obtained by means of the same procedure as the BTBO and BTBO:Nd^3+^ powders. The results of these measurements are presented in Figure 4.

As can be seen in the graph below, each of the lanthanides used also gave a decrease in the initial SHG level compared to undoped BTBO, but quantitatively, the results were different—only for Ho^3+^ ions was the result slightly weaker than for Nd^3+^ with identical dopant content. While there is no doubt that such pronounced changes in optical nonlinearity result from structural modifications within the BTBO crystal lattice, it is difficult (at this stage) to look for the cause, for example, in the difference in the ionic radii of the dopants used and any directional changes in this trend. For example, Ho^3+^ and Er^3+^ ions have very similar effective ionic radii (90.1 and 89.0 pm, respectively [58]), yet they yield two noticeably different results (8.5 and 15.4 × KDP, respectively). It seems much more probable that as a result of doping, some perturbations were brought to the distribution of electron energy density within atomic orbitals, which influenced the change of the nonlinear electric susceptibility tensor (as well as Judd–Ofelt parameters) and this effect is strictly dependent on both the matrix itself and the type and content of the RE^3+^ dopant [59,60,61]. In spectroscopic regions close to RE^3+^-absorption center resonances, significant changes in nonlinear coefficients are observed because the excitation of electrons and interaction with atomic orbitals may then be the most crucial [62,63,64]. Nevertheless, the results seen in Figure 4 remain very perspective and offer promising prospects for further research into the quantitative and qualitative influence of various RE^3+^ dopants on the optical nonlinearity of BTBO. At this stage of research, we selected three compounds for further studies: undoped BTBO and two doped ones—BTBO:Nd^3+^(0.5%) and BTBO:Nd^3+^(1.0%) (actually, the three with the strongest nonlinearity in the entire series).

**Figure 5:**
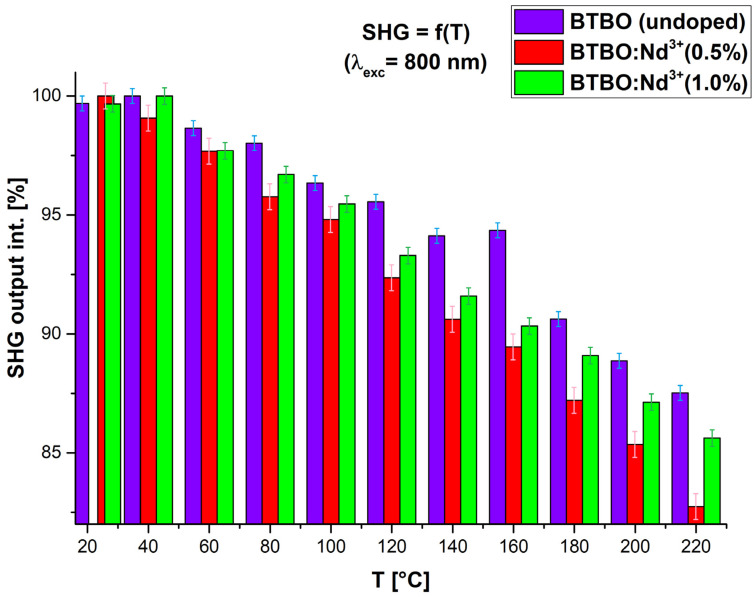
Relative individual temperature attenuation of the SHG in undoped and selected Nd^3+^-doped BTBO powders for the excitation source λ = 800 [nm].

In the second stage of SHG measurements, we examined the sensitivity of our selected BTBO and BTBO:Nd^3+^ powder samples to temperature changes within the range typical for laser device operation (from RT to slightly above 200 °C) in terms of their frequency doubling capability. It is implicitly expected that thermal expansion (described in the Section 3.3) changes the noncentrosymmetry of the medium, and additionally, increased phonon vibration amplitudes in the crystal lattice reduce the SHG efficiency (and other NLO effects). Since it is known from the literature that BTBO is transparent down to 350 nm [7] and the strongest decreases in optical nonlinearity can occur for the second-harmonic wavelengths closer to the fundamental absorption edge (which position is sensitive to RE^3+^ dopants [11]), we selected the source of λ = 800 nm (Ti:Al_2_O_3_ laser) as the fundamental wavelength of the 1ω in this experiment. This time, however, we did not use any comparative standards (i.e., KDP) nor did we focus on quantitative-comparative studies of absolute SHG signal between individual samples, but limited ourselves solely to the relative disappearance of this effect as a temperature function for each sample. The reasons for this decision were: a change of the optical excitation source, reconstruction of the system to the reflection mode, and a different scattering–absorption character of the tested samples compared to the KP method (related to their preparation, their optical sensitivity in the used system, and NLO coefficients dependency on 1ω). Additionally, in the case of the reflection mode used here, as well as the higher thickness of samples, it should be assumed that only a fraction of the total second harmonic signal was collected. Therefore, the tested samples behave in a manner more similar to that described by Aramburu et al. [65], and the actual description of the propagation of individual beams is much more complex than in the KP method. The results of temperature-dependent SHG measurements for the previously indicated samples are presented in Figure 5.

One should notice that the temperature regression of SHG differs for each investigated sample. Here we introduce some comments and explanations relevant to this phenomenon. First, the undoped sample exhibits the weakest temperature attenuation of NLO response (ca. 12.5% drop within the whole temperature range). The largest decrease was observed for BTBO:Nd^3+^(0.5%), exceeding 17%. The BTBO:Nd^3+^(1.0%) sample ranks intermediate (less than 15% decrease). Analyzing the entire series of these waveforms, it can be seen that the SHG signal remains relatively stable and at a similar level up to 40 °C, after which a gradual weakening of the optical response begins. While this decrease appears linear for both Nd^3+^-doped BTBO samples, the undoped sample appears to deviate from this pattern. Such differences between the doped and undoped BTBO samples may result from the presence of additional absorption centers in the former ones (more on this in Section 3.2), resulting from the introduction of Nd^3+^ ions into the structure. The literature has shown that these absorption centers also behave differently with varying crystalline temperatures [66,67]. It is emphasized that the increase in temperature causes an increase in the relaxation rate, which affects the spectral characteristics and decoherence processes [68]. Nevertheless, maintaining the SHG signal with 2ω frequency close to the absorption edge of the investigated materials at a temperature just above 200 °C, and at a level exceeding 80% of the value measured at RT, seems to be a satisfactory result. To estimate which and how strong absorption centers of Nd^3+^ ions could play a role here, UV-VIS spectroscopic studies were carried out for BTBO and BTBO:Nd^3+^ powders, as described in the next section.

### 3.2. Absorption Studies and Determination of the Energy Band Gap of BTBO:Nd^3+^

**Figure 6:**
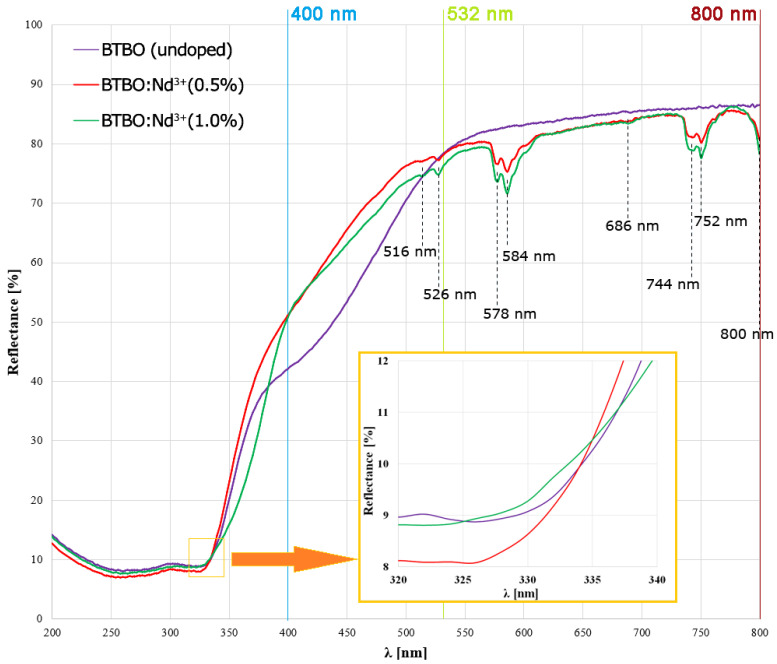
DRS of undoped and Nd^3+^-doped BTBO samples, along with Nd^3+^-assigned absorption bands marked. The inset shows a zoom for the absorption edge/threshold region. The graph has also been marked with the spectral lines characteristic of SHG of the Ti:Al_2_O_3_ and Nd:YAG lasers (400 and 532 nm, respectively) and the fundamental wavelength of Ti:Al_2_O_3_ (800 nm).

Diffuse reflectance spectra (DRS) were recorded for the obtained powder materials: undoped BTBO and selected Nd^3+^-doped ones. This type of spectrum was most suitable for analysis, primarily due to the dispersive nature of the obtained powders. The obtained spectra are presented in the summary graph in Figure 6.

On the spectra of BTBO:Nd^3+^(0.5%) and BTBO:Nd^3+^(1.0%) samples, one can see peaks in the wavelength range from 515 to 800 nm, while the curve of undoped BTBO is essentially continuous in its whole course. These peaks detected for doped samples are assigned to absorption centers characteristic of Nd^3+^ ions, as listed in Table 2. Importantly, the higher the Nd^3+^ concentration in the sample, the bigger the peaks that were observed. Within the literature, the mentioned absorption centers have been described in detail, i.e., by Ramos-Lara et al. [69]. The bands located at 526, 578, and 584, as well as 744 and 752 nm, are attributed to the f-f transitions from the ground state (^4^I_9/2_) to the higher excited states (^4^G_7/2_; ^4^G_7/2_ + ^4^G_5/2_; and ^4^F_7/2_ + ^4^S_3/2_), respectively [70,71,72]. The presented results indicate that substituting for the Bi^3+^ ions in BTBO with Nd^3+^ ions was successful. It is also important to add that at the region close to the detection limit of the used spectrophotometer, around 800 nm, one of the strongest absorption bands for Nd^3+^ (^4^F_5/2_ + ^2^H_9/2_) was partially recorded. This is important because this crucial absorption line is clearly split and then efficiently covers the 1ω frequency used in the temperature-dependent SHG measurements described in this work. As a consequence, the fundamental photons with a frequency of 1ω corresponding to λ = 800 nm are not entirely used for SHG, but partially participate in other radiant energy transfers associated with transitions (I9/24→F5/24+H9/22), and this is the basis for the weaker NLO response for Nd^3+^-doped BTBO (as seen in Figure 5). This phenomenon was previously observed in numerous reported studies [73,74,75,76]. Detailed studies on the mechanisms of radiative and non-radiative transitions, interionic transitions, and luminescence in the VIS and NIR for BTBO:Nd^3+^ in the dopant concentration range up to 10 at.% have been published by our group earlier [9]. Since the main emission bands of Nd^3+^ ions may occur in NIR, the radiative effects of this strong absorption are not observable in any other direct form (despite weakening SHG near absorption edge) in the visible region [77]. However, despite the deeper absorption peaks originating from neodymium for BTBO:Nd^3+^(1.0%), in this case, the SHG weakening for excitation λ = 800 nm is not stronger than for BTBO:Nd^3+^(0.5%), but the reasons for this can hardly be assumed to be dopant concentration quenching [78]. In the DRS spectrum (Figure 6), it is also noticeable that while undoped BTBO and BTBO:Nd^3+^(0.5%) do not have deepened absorption at ca. λ=532 nm (2ω for the Nd:YAG excitation source) the band around 526 nm starts to widen and deepen for BTBO:Nd^3+^(1.0%)—suggesting an intensification of this effect with further doping and thus a weakening of SHG in this area (as shown in Figure 3). For the undoped BTBO sample the reflectance drop is steeper just below 525 nm, and it reaches about 10% lower when closer to the 400 nm threshold, compared to the Nd^3+^-doped ones.

**Figure 7:**
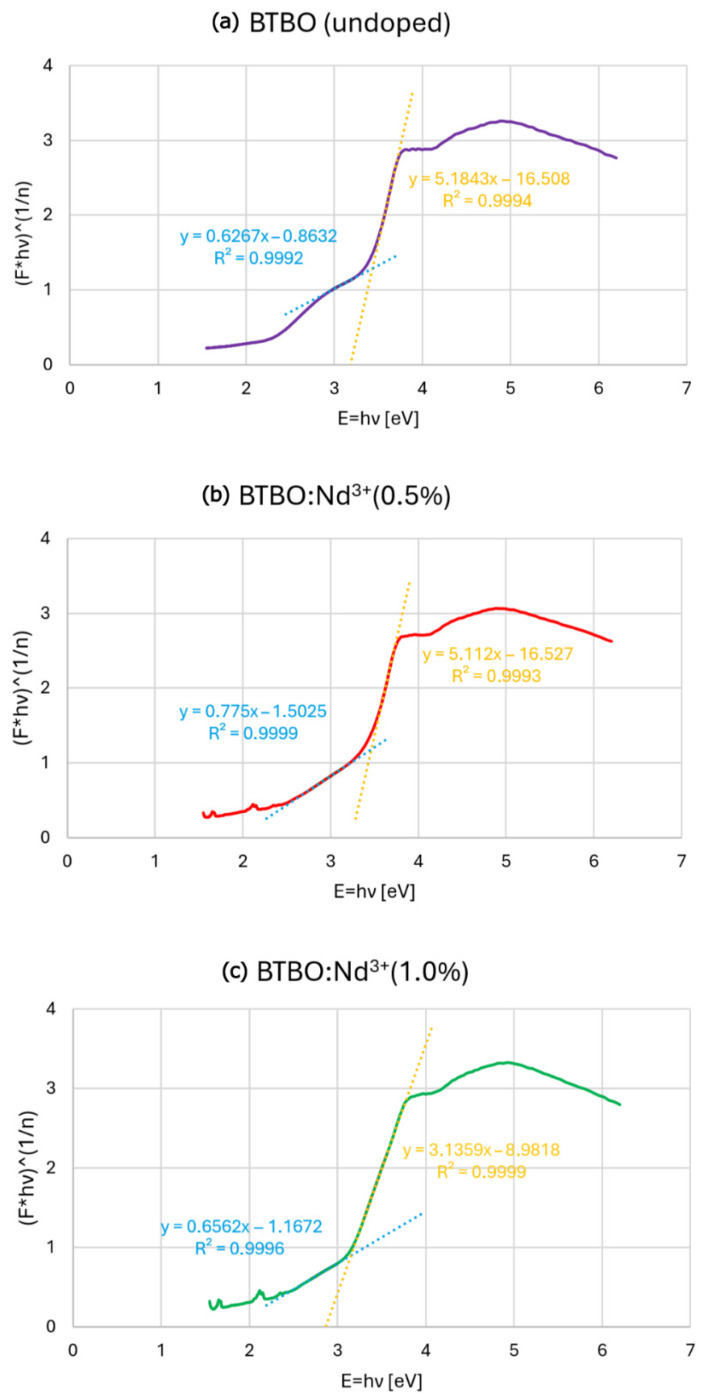
Transformed DRS plots of undoped (**a**) and Nd^3+^-doped (**b**,**c**) BTBO samples. The determination of the BG is shown on the graphs. The BG value is the x-component intersection point of dotted lines, where the blue dotted line is a fitted baseline, the yellow one is a fitted slope line, and the continuous purple/red/green line is a Tauc plot of corresponding analyzed samples, respectively.

The details of the BG determination method have been described elsewhere [45]. Briefly, the Kubelka–Munk function was applied to transform the diffuse reflectance spectra to values proportional to the absorbance. Based on this, the Tauc plot ((F*hν)^(*1*/*n*)^) in the function of *hν* was drawn (Figure 7a–c). In the equations employed, the *hν* is the incident photon energy, and *n* is the exponent of the determined electron transition. The corresponding BGs for undoped and selected Nd^3+^-doped BTBO powders are presented in Table 3.

At this point, it is worth underlining that BTBO has its BG of indirect type [16,17]. For this reason, we do not convert the obtained BG values to their corresponding wavelengths from the fundamental absorption edge. Additionally, one can notice from the inset in Figure 6 that these edges are closely overlapped. For undoped BTBO, we obtained a result identical to that reported by Song et al. [11], where a very similar method was used. In that case, for Tb^3+^ dopant concentrations ranging from 1 to 15 at.%, the corresponding BGs then covered the range of 3.14–3.24 eV. At this stage, it is difficult to predict the directional relationship between Nd^3+^ concentration and BG dimension, but based on the results in Table 3, it can be assumed that the absorption of the BTBO matrix may be even more sensitive to the lesser amount of neodymium dopant than was the case for terbium of a wider concentration range. There are some research studies that suggest the influence of the crystallite size of the dielectric materials on the behavior of BG [79,80,81]. However, as will be shown in the next chapter of the research (Section 3.3), the average sizes of the obtained BTBO and BTBO:Nd^3+^ crystallites are of the same order of magnitude. Also, the effect of quantum confinement on BG is questionable here (despite the existence of crystallites below 100 nm), because it usually leads to an increase, not a decrease, of BG [82].

### 3.3. Anisotropy of Thermal Expansion in BTBO:Nd^3+^

**Figure 8:**
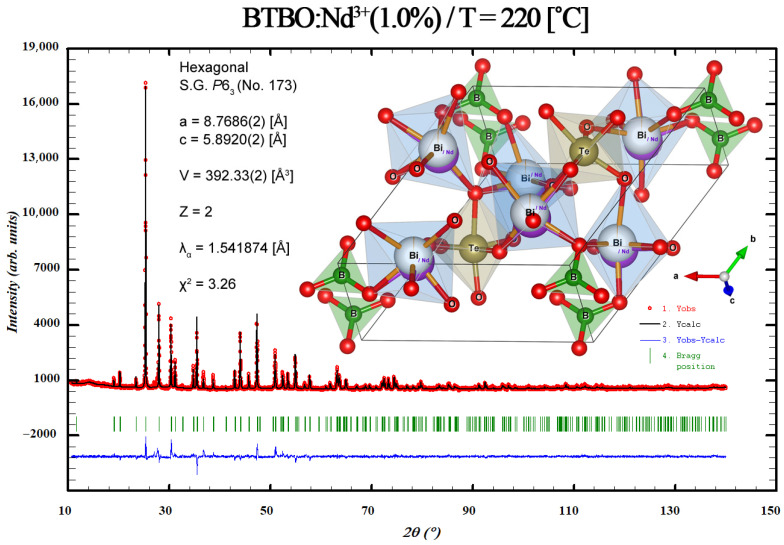
An exemplary, experimental XRD powder diffraction pattern along with a complete Rietveld refinement plot and refined structure of the Nd^3+^-doped BTBO unit cell at an elevated temperature.

As mentioned in Section 2.2.4, Rietveld refinement was performed for a complete set of diffraction patterns from all tested samples, which were measured in the temperature range from ca. RT to 220 °C. The reference (initial) BTBO structure was taken from the CIF standard file provided by Xia et al. [2] with the database reference number COD No. 96-412-5286. Regardless of the tested sample and measurement temperature, the refinement parameter χ^2^ did not exceed the value of 3.97. An example of such an XRD pattern of the BTBO:Nd^3+^(1.0%) after structure refinement, together with its 3D projection, is presented below in Figure 8.

**Figure 9:**
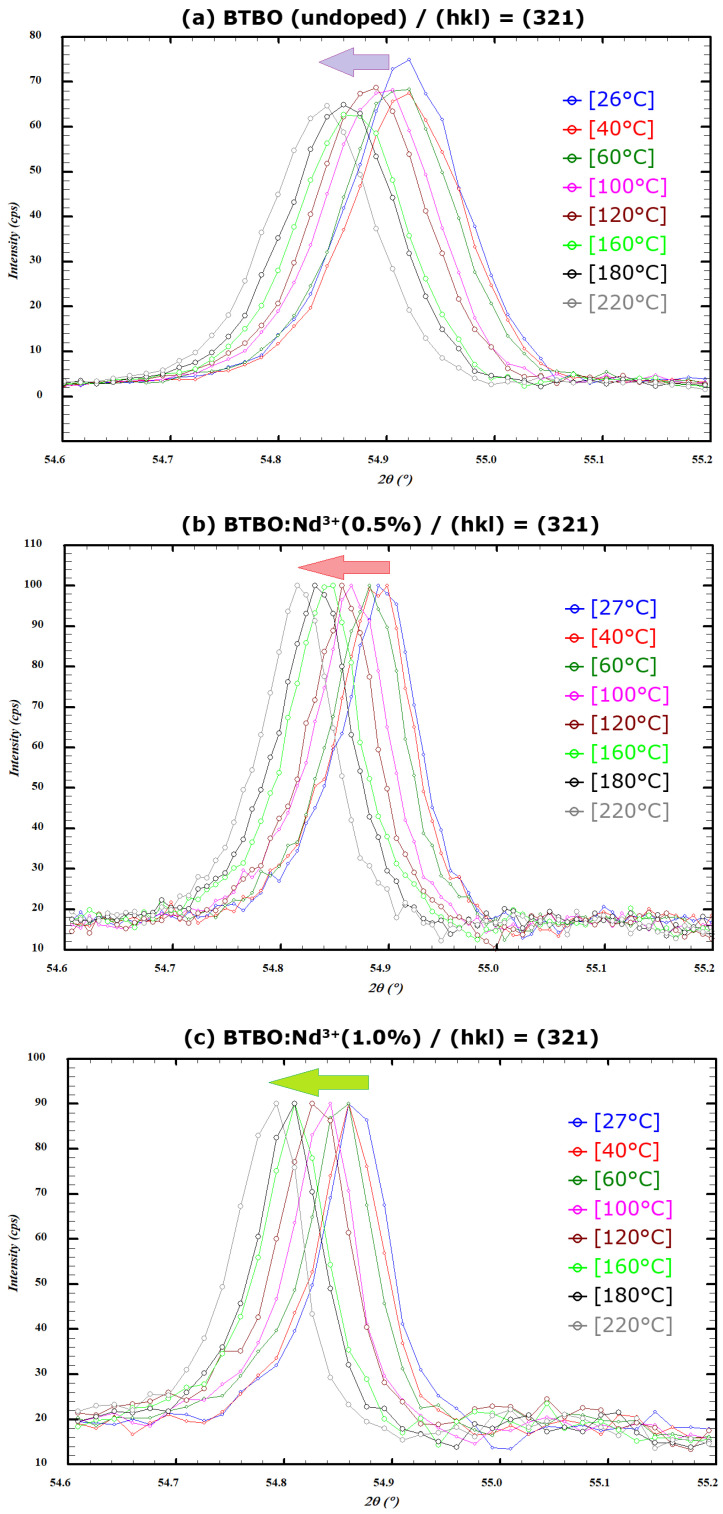
Temperature-dependent Bragg position migration of the (321) plane for undoped (**a**) and Nd^3+^-doped (**b**,**c**) BTBO powders. The direction of the arrows indicates shifts consistent with increasing measurement temperature.

One can observe in Figure 9 the behavior of a selected lattice plane (here, (321)) and its shifts (Bragg position changes) as a function of temperature for undoped and selected Nd^3+^-doped BTBO powders. The illustrated tendency (moving the (hkl) line towards lower diffraction angles) is typical for lattice planes of materials exhibiting positive thermal expansion anisotropy [83]. Table 4 provides a complete list of the lattice constants of undoped and selected Nd^3+^-doped BTBO powders for all measured temperatures.

In the next step, the temperature profiles of each lattice constant were approximated for all three investigated samples in order to obtain the best model of the temperature-dependent function. For this purpose, we performed fitting using a function based on a polynomial close to the Lagrangian theory [84,85], like that described in the work of Mosafer et al. [86]. Since it is clear from Table 4 that the largest temperature increases of the unit cell occur in each case for constant *a* and volume *V*, an approximation function of the following type was chosen for these parameters:

(3) $$L_{a,V}\left(T\right)=A+B*T+\frac{C}{T},$$

**Figure 10:**
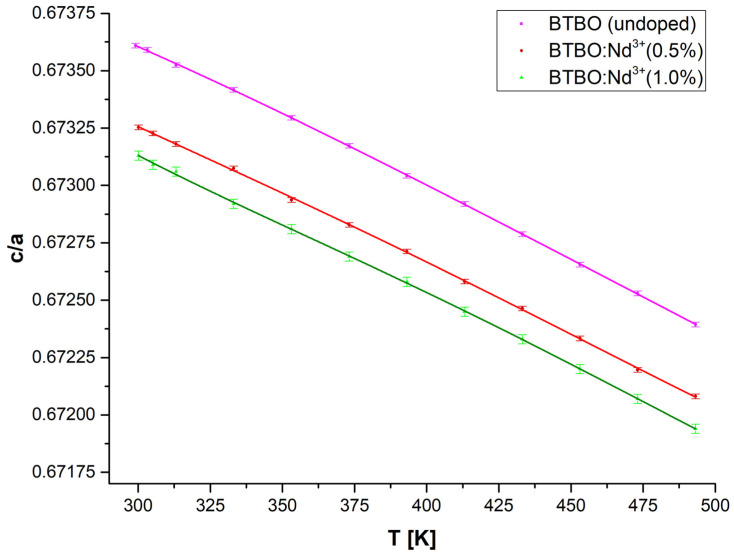
Temperature course of the *c*/*a* ratio and its fitting for undoped and Nd^3+^-doped BTBO samples.

Whereas in the case of the lattice constant *c*, for which (in each sample) very small increases of this parameter were observed (of the order of 0.0001, less often 0.001 Å), and so a linear approximation was used. Additionally, an approximation of the (*c*/*a*) ratio was also performed (see Figure 10) using a fourth degree polynomial function.

After finding the rest of the coefficients included in the mentioned equations, the main functions were differentiated with respect to temperature, and the following operator was used to determine the relative thermal expansion coefficients (TEC) *α_y_*(*T*) (where *y* = *a*, *c*, *V*):

(4) $$\alpha_{y}\left(T\right)=\frac{dy(T)}{dT}\cdot \frac{1}{y(T)}$$

**Figure 11:**
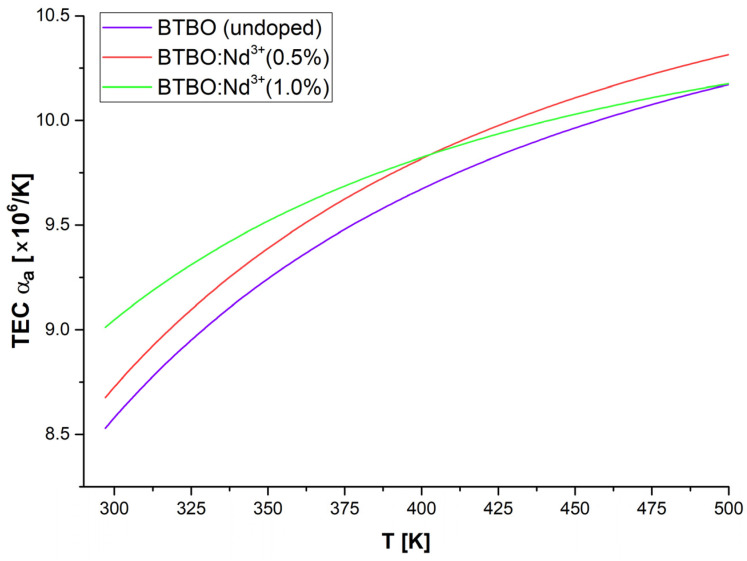
Experimentally calculated curves of the relative thermal expansion coefficient along the *[100]* direction for undoped and Nd^3+^-doped BTBO samples.

**Figure 12:**
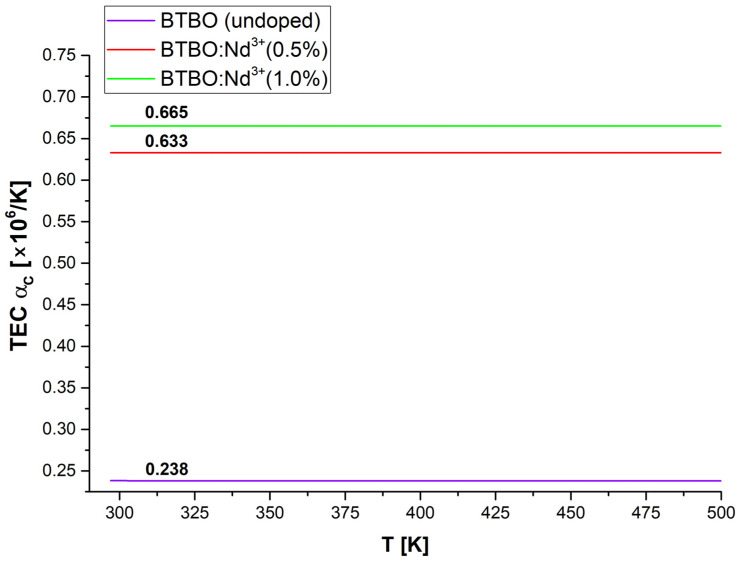
Experimentally calculated curves of the relative thermal expansion coefficient along the *[001]* direction for undoped and Nd^3+^-doped BTBO samples.

**Figure 13:**
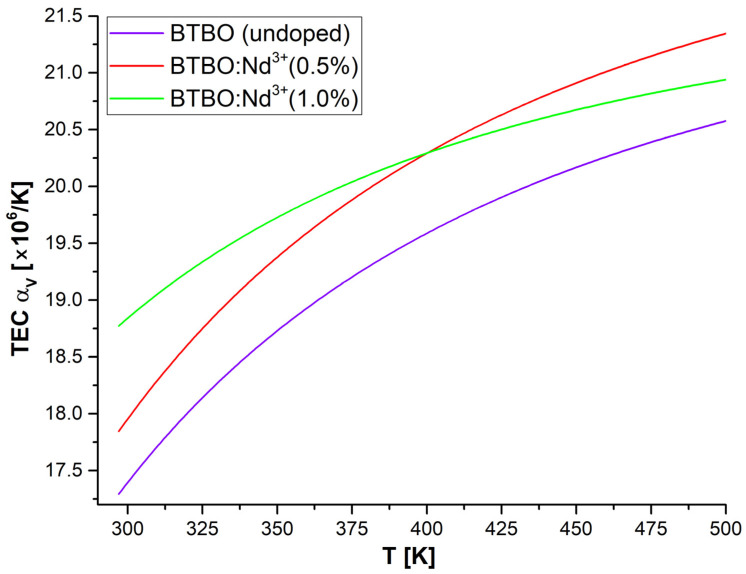
Experimentally calculated curves of the relative volumetric thermal expansion coefficient for undoped and Nd^3+^-doped BTBO samples.

The series of figures below (Figure 11, Figure 12 and Figure 13) shows the corresponding graphs, *α_a_*, *α_c_*, and *α_V_*, as functions of temperature.

After a detailed analysis of each of the TEC coefficient curves presented above (and supplementary with the previously presented Table 4 and Figure 10), a number of key conclusions can be written summarizing the phenomenon of anisotropy of the thermal expansion of the studied BTBO and BTBO:Nd^3+^ powders. First, the thermal expansion of parameters *a* and *c* occurs in a strongly disproportionate manner, less noticeably along the principal crystallographic direction *[001]*, which is parallel to the polar axis in the *P*6_3_ space group. This effect is essentially independent of the doping content of the tested samples (this is visible in Figure 10 as almost identical slopes of each curve). Secondly, however, depending on the dopant concentration, the initial lattice constants (at RT) are modified. Here, the empirical assumption related to the relationship between the ionic radii of bismuth and neodymium meets good agreement, meaning that smaller neodymium (98.3 pm) substituting the positions of bismuth (103 pm) causes a decrease in the unit cell volume, and this effect is stronger for a higher content of Nd^3+^ ions (same trend as for Tb^3+^ reported in [11]). Third, TEC *α_a_* is a nonlinear function of temperature, and its volumetric counterpart TEC *α_V_* (strongly dependent on TEC *α_a_*) represents an even stronger nonlinearity. Additionally, TEC *α_a_* and TEC *α_c_* satisfy the empirical relationship TEC *α_V_* =2·TEC *α_a_* + TEC *α_c_* for the hexagonal class quite well in each case (best for undoped BTBO) [87]. Fourth, in the temperature range up to about 400 K (ca. 127 °C), the relative expansion of the BTBO matrix is proportional to the Nd^3+^ dopant content (undoped BTBO experiences the weakest expansion). However, above 400 K, this trend changes within doped samples, where cross-section occurs on plots in Figure 11 and Figure 13**,** and BTBO:Nd^3+^ (0.5%) dominates over the other two materials. Fifth, the thermal expansion along the *[001]* direction is negligible but almost constant with temperature (TEC *α_c_* ~ constant), regardless of the analyzed material. However, the TEC *α_c_* dimension is strongly dependent on the dopant content (0.5 at.% neodymium in the BTBO structure increases this coefficient by more than 2.5 times compared to undoped). After all, TEC *α_a_* and TEC *α_c_* differ by about an order of magnitude. We suggest that it is the negligible thermal expansion along the polar axis of BTBO that allows (in the first instance) for the counteracting of the acentricity perturbation of the unit cell during heating and, as a result, the ability to maintain a quite accessible SHG signal (coming from volumetric particles) within the given temperature range (even for 2ω frequency close to the fundamental absorption edge). The trend in the TEC *α_V_* curves (Figure 13) fits well with the SHG = f(T) attenuation plots (Figure 5) from 400 K upwards in each case, which confirms the above-mentioned assumption.

Interestingly, in the work of Dong et al. [7], the authors reported negative thermal expansion of undoped BTBO along the *[001]* direction, but using a completely different measurement method (dilatometric, on 1.0 mm thick single-crystal wafers with (100) and (001) orientations). All results from the work described are contradictory to the current results of TEC parameters obtained by means of the diffraction method (additionally, the authors of [7] have used average values, not temperature functions, which makes it completely impossible to compare their results and our own). The diffraction method considers a certain phenomenon at the atomic level, while the dilatometric method considers it at the macroscale. The influence of internal stresses or defects may cause differences in these results, as a single-crystal sample may behave differently under the influence of external and intrastructural stresses, a distinction which may not be observed in the case of powder samples, especially those containing micro- and nano-fractions of particles. In recent years, this phenomenon has been known and described, and is attributed to differences in measurement methods, expansion anisotropy, stress effects, and microstructural processes. Another cause of such occurrence may be the presence of domains (but not in the case of BTBO and its non-ferroelectric space group *P*6_3_, despite being a polar group). Publications documenting such cases and discussing their causes can be found in the literature [88,89]. Of course, possible differences in TEC parameters between single crystals and polycrystals of the same compound are nothing extraordinary, but in the described case, the nature of this anisotropy is very unusual.

When it comes to a comparison of known (host) matrices for SFD applications and their TEC coefficients, the powders studied here, BTBO and BTBO:Nd^3+^(0.5%) (with the strongest NLO response for the Nd:YAG source), are very promising, among some others or at least comparable ones (see Table 5 below). They have lower selected TEC coefficients than, e.g., La_2_CaB_10_O_19_ and LiNbO_3_ (and its TEC *α_c_* is at least an order of magnitude lower compared to all listed), which makes the BTBO potentially desirable as a material for SFD (if RE^3+^-doped) device applications.

The previous studies and the evolution of the lattice constants in BTBO and BTBO:Nd^3+^ investigated in this work, as well as their TEC coefficients, did not show any discontinuities in courses that could indicate the possibility of the occurrence of any phase transition (either first-order or second-order) within the temperature range from RT to 220 °C. As described in the next section of this work (Section 3.4), the range of higher temperatures, up to the melting point of the synthesized powders, was also investigated in these terms (with emphasis on the possible influence of the Nd^3+^ dopant in triggering such transformations).

### 3.4. Thermal Stability of BTBO:Nd^3+^

**Figure 14:**
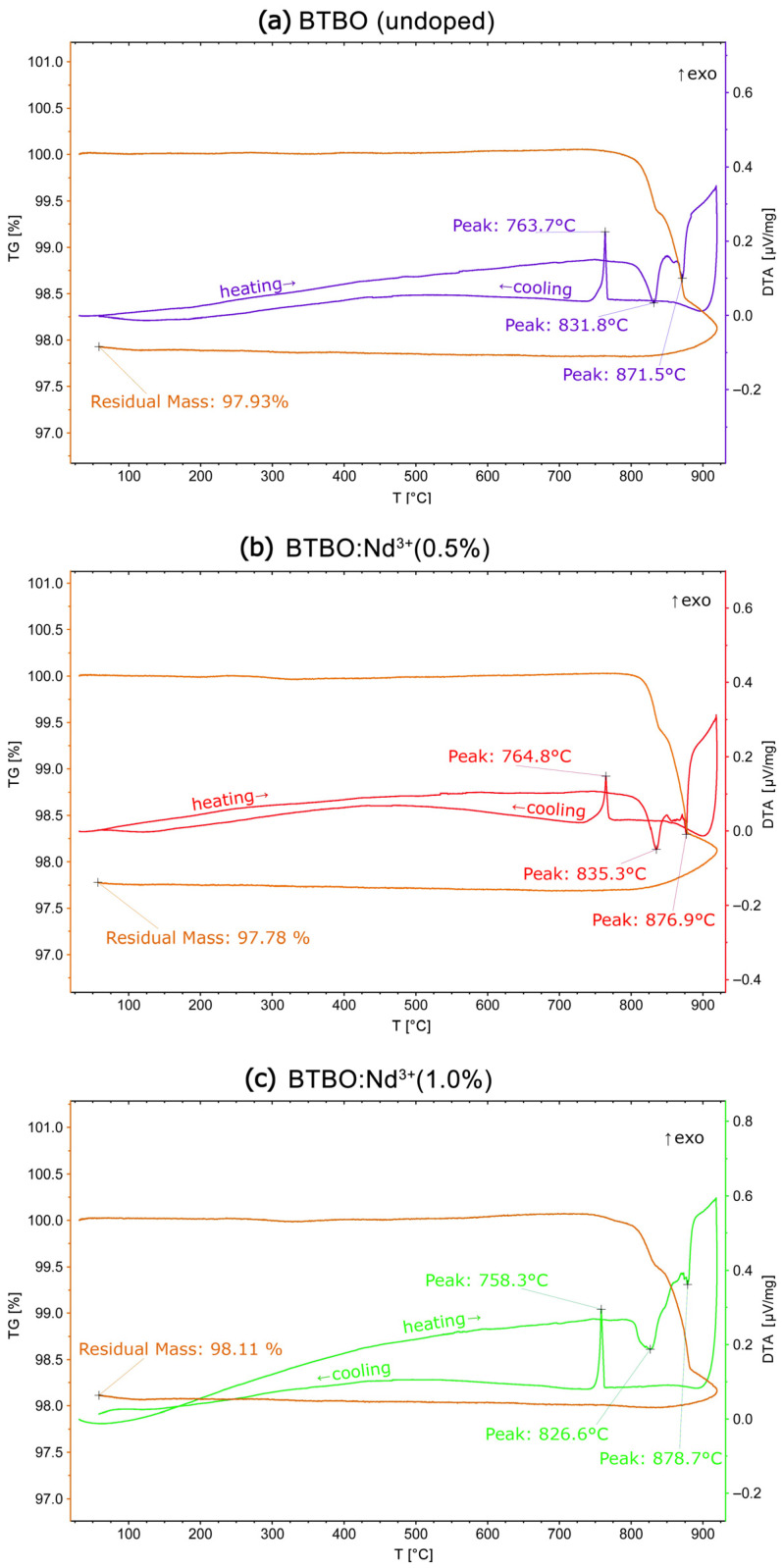
DTA-TG curves for undoped (**a**) and Nd^3+^-doped (**b**,**c**) BTBO samples.

In Figure 14a–c below, the collective thermogravimetric plots (DTA-TG) for undoped and selected Nd^3+^-doped BTBO powders are presented.

All presented DTA-TG curves do not differ substantially from those published in previous works [1,7]. No significant effect of the Nd^3+^ dopant content or its concentration on the melting temperature of BTBO:Nd^3+^ was observed. The melting process in each run begins slightly above 800 °C, and the sample mass remains stable up to this point. The incongruent melting behavior was confirmed in the control measurement for undoped BTBO and then for BTBO:Nd^3+^. This is evidenced by two sharp endothermic peaks—the first in the range of 826–836 °C, and the second in the range of 871–879 °C. Between these thermal effects, partial thermal decomposition of the samples occurs, which is associated with mass loss (less than 2.5%, with 1% accuracy). This decomposition results from the chemical instability of the incongruent melting products, and most likely, boron and/or tellurium compounds may undergo partial volatilization [95,96,97]. The issue of chemical composition in incongruently melted BTBO was reported by Daub et al. [1], who also pointed to the loss of oxygen accompanying this decomposition, resulting from the reduction of tellurium from the +6 to +4 oxidation state. After melting, recrystallization of the tested samples always occurs below 765 °C. Both the positions of the melting peaks and those of recrystallization differ slightly depending on the dopant level. These differences coincide with the maximum permissible measurement uncertainty of the used apparatus. For this reason, it is difficult to determine a directional relationship, for example, between the BTBO:Nd^3+^ doping level and its melting temperature, from the obtained DTA-TG measurement results. However, the study clearly confirmed the absence of any phase transitions in the range from RT up to its incongruent melting point. This allows us to consider the tested BTBO:Nd^3+^ materials as temperature-stable ones within the region of application of typical optoelectronics devices. Compared to the melting points of such SFD matrices as LiNbO_3_ or YAl_3_(BO_3_)_4_ (1260 and 1280 °C, respectively), BTBO:Nd^3+^ are not such high-melting-point materials [57,98].

### 3.5. Distribution of Neodymium Dopant in BTBO:Nd^3+^

**Figure 15:**
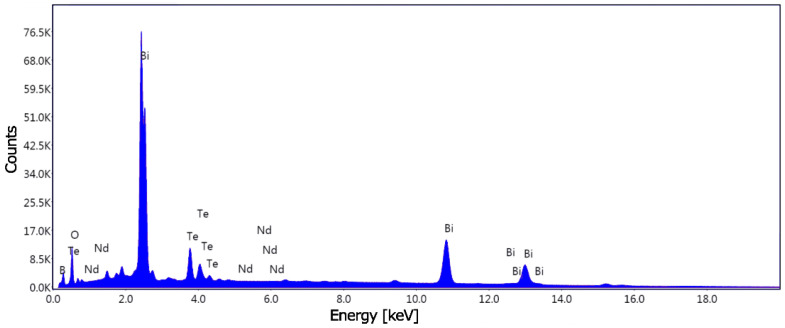
An exemplary EDX spectrum for the BTBO:Nd^3+^(1.0%) sample.

The EDX microelemental analysis of the investigated samples allowed for the determination of the element content for BTBO:Nd^3+^ samples. In Figure 15**,** an exemplary spectrum for the selected BTBO:Nd^3+^(1.0%) powders is presented.

Discrepancies between experimental and actual elemental concentrations in such compounds are a common problem in EDX measurements, especially when they consist of light elements (boron, oxygen) and heavy ones (bismuth, RE metals), as is the case with BTBO. We also observed these discrepancies in our present study. This problem is exacerbated when the issue concerns not the stoichiometric elements but dopants introduced in very small amounts into the crystalline matrix. We have previously identified and explained the exact causes of these discrepancies in detail in our works [13,18,99,100]. In the paper published by Piekara et al. [100], an approach was presented that allows us to estimate the actual dopant concentration based on the ratio of the dopant element content to the substituted one. Using this model, we later examined the consistency of the expected and experimental dopant concentrations for BTBO:Yb^3+^ compounds [13], obtaining satisfactory results. Below, in Table 6, we present the results of these calculations for the BTBO:Nd^3+^(0.5%) and BTBO:Nd^3+^(1.0%) systems, based on the raw EDX measurements performed previously in the work of Zhezhera et al. [9].

As can be seen from Table 6, the best agreement was obtained for an Nd^3+^ content of 1.0 at.%. For 0.5 at.%, the measured result is twice as low, but within the same order of magnitude as the expected value. Furthermore, the ratio of lower to higher concentrations is preserved. Because of the measurement resolution of EDX, the associated measurement uncertainties, inhomogeneous morphology of studied powders, the local nature of information, and a number of other artifacts resulting from the measurement method itself, the final result can still be considered satisfactory for such low doping Nd^3+^ levels.

**Figure 16:**
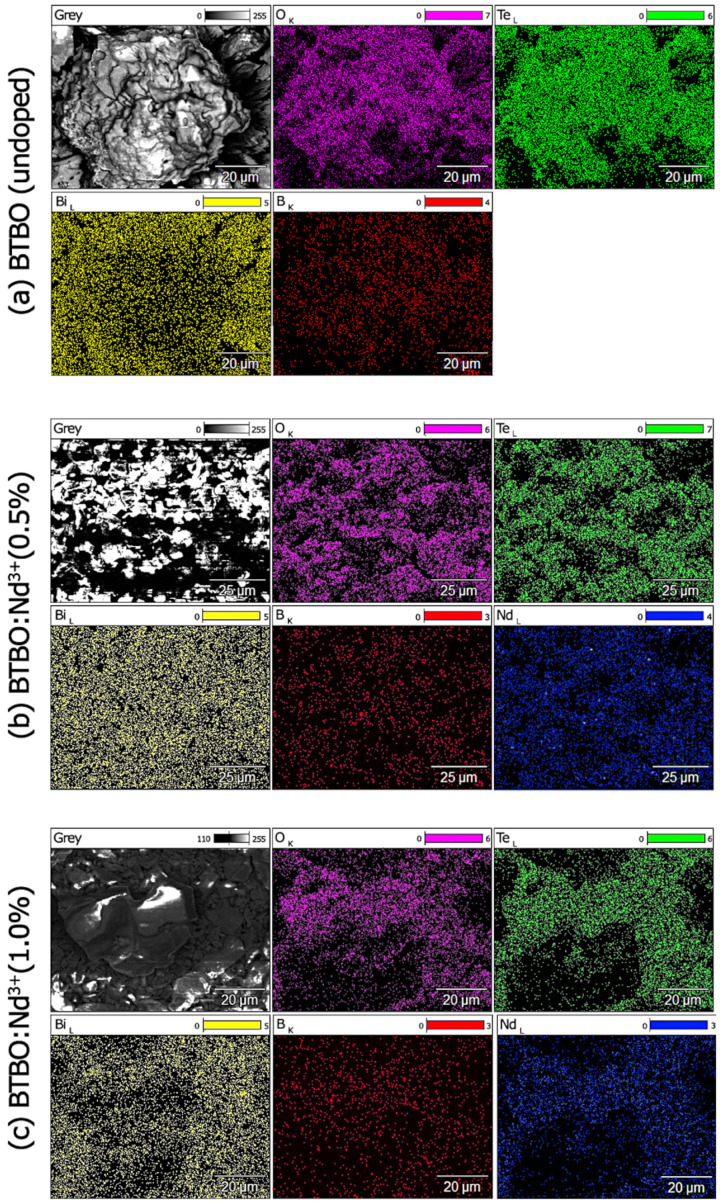
EDX mapping for the presence of individual elements for undoped (**a**) and Nd^3+^-doped (**b**,**c**) BTBO samples.

Figure 16 presents EDX maps of the elements constituting the BTBO and BTBO:Nd^3+^ powders. This imaging focuses specifically on analyzing the homogeneity of the distribution of the dopant element (Nd) relative to the substituted element (Bi). Each element is assigned a specific color mapping (the window marked “Grey” represents the original SEM image from which the elemental information was derived). Analysis of individual images indicates that in each case the dopant distribution is homogeneous, with no significant distortions in the signal from any element. The modified Pechini method, which was used to synthesize the BTBO and BTBO:Nd^3+^ powders, guarantees a homogeneous (at the atomic level) distribution of the elements already included in the complex oxides at the preparation stage. This is possible because the intermediate preparatory step of these syntheses is performed using a liquid phase of a homogeneous solution of precursors, which can essentially be mixed at the atomic level. This, in turn, influences the results obtained.

### 3.6. Morphology of BTBO:Nd^3+^ Nanofraction

**Figure 17:**
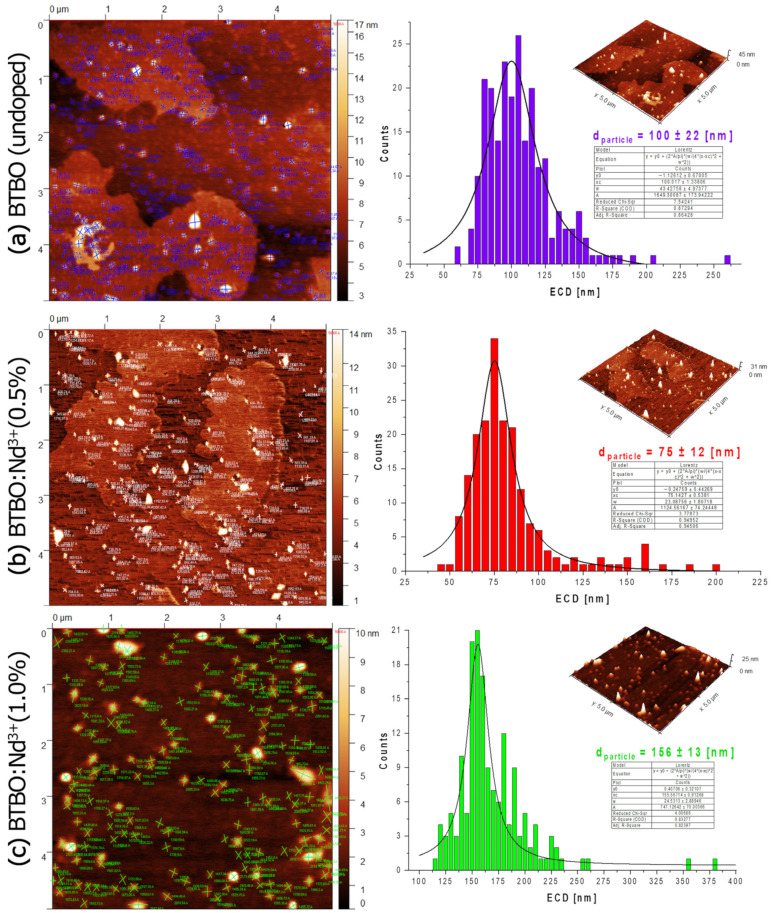
AFM images (2D phase and 3D topography) and ECD distributions of undoped (**a**) and Nd^3+^-doped (**b**,**c**) BTBO circa-nanometric particles obtained from the finest powder fractions by means of sonication and sedimentation after syntheses.

Below, in Figure 17a–c, a complete AFM topography analysis of fractionated BTBO and selected BTBO:Nd^3+^ powder particles is provided, along with their dimensioning and an analysis of their average equivalent circular diameter (ECD) size distribution.

Each ECD distribution represents a well-fitted Lorentz-type shape. AFM topography of the examined powders revealed their quasi-nanostructure. Due to the microscope’s ability to visualize nanomaterials, such as powders or flake graphene, in three dimensions [101,102], the focus was on the smallest fractions of the synthesized powders. The study revealed a homogeneous dispersion of individual nanoparticles of the material. It is worth underlining that the dispersion was maintained regardless of the sample variant. An attempt was made to determine the average particle size of the powders. The study indicated slight differences between the materials. The smallest fraction was found to be of d_particle_ = 75 ± 12 nm. The differences in the average sizes of these particles for all the tested materials may result from purely stochastic factors of a given sample preparation attempt. Similar standard deviations of these diameters indicate a high degree of uniformity in the synthesized particles. It can be seen that the average particle sizes of these fractions are generally several dozen nanometers larger (and in the case of 0.5 at.% Nd^3+^, they are even very close), compared to the average crystallite sizes estimated in Table 4. However, it should be remembered that these are the dimensions of two other coexisting entities (because these particles consist of crystallites). It should also be added that AFM allows for direct observation of these particles, while the XRD calculations provide indirect and estimated results. Nevertheless, even obtaining a single result like the determination for 0.5 at.% Nd^3+^ can be considered a very satisfactory separation procedure for nanofractions. Most of the visualized particles, originating from the deagglomerated and separated fraction, seem to be of the spheroidal (ovoid-like) shape. Thanks to the self-evaporation of a liquid carrier from the filtrate at RT, the effect of aggregation is reduced to a minimum. During the AFM measurements, no random particle displacements over the scanned surface were observed, indicating that the BTBO and BTBO:Nd^3+^ particles show proper adhesion to the substrate.

## 4. Conclusions

The study reveals that neodymium (Nd^3+^) doping in Bi_3_TeBO_9_ (BTBO) powders significantly influences their nonlinear optical properties, particularly second-harmonic generation (SHG). At room temperature the undoped BTBO powders exhibit a strong SHG efficiency, consistent with prior reports. The introduction of a small Nd^3+^ content (around 0.5 at.%) enhances SHG intensity by over 15%, likely due to subtle stoichiometric changes improving the material’s acentricity. However, higher doping concentrations reduce SHG intensity, though, even at 5 at.%. The nonlinear response remains significant compared to other borates.

Lanthanide doping with other RE^3+^ ions (Tm, Ho, Pr, Er) at 1 at.% lowers SHG relative to undoped BTBO but retains promising values. The optical nonlinearity changes are attributed not just to ionic size differences but to electron density distribution perturbations around atomic orbitals, affecting nonlinear susceptibility. Temperature-dependent SHG studies demonstrate a stable SHG response up to 40 °C, followed by gradual attenuation, with Nd-doped samples showing greater thermal sensitivity, likely due to Nd^3+^ absorption centers influencing relaxation processes. Still, over 80% of initial SHG is retained near 200 °C, indicating thermal robustness for device operation.

Diffuse reflectance spectroscopy confirms successful Nd^3+^ incorporation, revealing characteristic f-f absorption bands. Corresponding band gap measurements show slight variations with doping but remain in the indirect gap range, consistent with BTBO’s known electronic structure. Crystallite sizes are comparable across samples, suggesting that band gap changes derive from doping effects rather than quantum confinement.

Temperature-dependent X-ray diffraction and Rietveld analysis show anisotropic thermal expansion predominantly along the hexagonal a-axis, and disproportionately weaker along the polar c-axis—a behavior partially affected by Nd content. Thermal expansion coefficients of BTBO:Nd^3+^ are favorable compared to other self-frequency doubling (SFD) crystals, and no phase transitions occur up to the melting point, as supported by differential thermal analysis.

Thermogravimetric studies confirm the incongruent melting behaviors typical of BTBO unaffected by neodymium doping. Elemental analysis via EDX mapping demonstrates uniform Nd distribution at the atomic scale, due to the advantages of the applied modified Pechini synthesis method. Atomic force microscopy reveals quasi-nanostructured powders with well-dispersed, spheroidal nanoparticles, primarily round, even at 75 nm in diameter, without aggregation effects.

Overall, Nd^3+^-doped BTBO powders present a balanced multi-functional platform with enhanced nonlinear optical properties, thermal stability, and structural integrity, making them promising candidates for SFD devices and potential applications in optoelectronics and bio-photonics devices.

## Figures and Tables

**Table 1:** Summary of the nonlinear optical responses of various borate crystals, compared to the current experiment (each for fundamental wavelength of λ = 1064 [nm]).

Material	SHG [KDP]	Ref.
Bi_3_TeBO_9_	20	[2]
Bi_3_TeBO_9_	20.8	This work
Bi_2.985_Nd_0.015_TeBO_9_	24	
Bi_2.97_Nd_0.03_TeBO_9_	9.2	
β-BaB_2_O_4_	4	[52]
γ-Be_2_BO_3_F	1.6	
Bi_2_ZnOB_2_O_6_	0.585	[18]
BiB_2_O_4_F	10	[53]
CsLiB_6_O_10_	2.5	[54]
KBe_2_BO_3_F_2_	1.2	[52]
La_2_CaB_10_O_19_	2	[55]
LiB_3_O_5_	3	[56]
NH_4_B_4_O_6_F	3	[52]
YAl_3_(BO_3_)_4_	6.0	[57]
α-BiB_3_O_6_	8	[53]
δ-BiB_3_O_6_:RE^3+^	4–6	[51]

**Table 2:** Characteristic electronic transitions of neodymium ions with their typical spectral ranges and experimentally determined spectral positions for Nd^3+^-doped BTBO powders.

Transition Type	Spectral Range [nm]	Band(s) Detected [nm]	Character
I9/24→F5/24+H9/22	~800–820	800	Strong
I9/24→F7/24+S3/24	~740–760	744; 752	Strong
I9/24→F9/24	~680–690	686	Very weak
I9/24→G5/24+G7/22	~575–590	578; 584	Strong
I9/24→G9/24+G7/24	~515–530	516; 526	Weak

**Table 3:** Band gap values of the analyzed undoped and Nd^3+^-doped samples.

Compound	BTBO (Undoped)	BTBO:Nd^3+^(0.5%)	BTBO:Nd^3+^(1.0%)
**Band Gap**	3.43	3.46	3.15
**(±0.03 [eV])**			

**Table 4:** Complete summary of the unit cell parameters of undoped (**a**) and selected Nd^3+^-doped (**b**,**c**) BTBO powders obtained from Rietveld refinement for the whole temperature range from RT to 220 °C. The estimated crystallite size (**D_cryst._**) was determined at RT.

Compound	T [°C]	T [K]	a [Å]	c [Å]	c/a	V [Å^3^]	D_cryst._ [nm]
(**a**) **BTBO** **(undoped)**	26	299.15	8.7523(1)	5.8956(1)	0.67361(1)	391.11(1)	59(1)
	30	303.15	8.7525(1)	5.8956(1)	0.67359(1)	391.13(1)	
	40	313.15	8.7533(1)	5.8956(1)	0.67352(1)	391.20(1)	
	60	333.15	8.7549(1)	5.8957(1)	0.67342(1)	391.35(1)	
	80	353.15	8.7563(1)	5.8956(1)	0.67329(1)	391.47(1)	
	100	373.15	8.7582(1)	5.8958(1)	0.67317(1)	391.65(1)	
	120	393.15	8.7598(1)	5.8957(1)	0.67304(1)	391.79(1)	
	140	413.15	8.7615(1)	5.8958(1)	0.67292(1)	391.94(1)	
	160	433.15	8.7632(1)	5.8958(1)	0.67279(1)	392.10(1)	
	180	453.15	8.7649(1)	5.8958(1)	0.67265(1)	392.25(1)	
	200	473.15	8.7667(1)	5.8959(1)	0.67253(1)	392.42(1)	
	220	493.15	8.7684(1)	5.8958(1)	0.67239(1)	392.57(1)	
**Compound**	**T [°C]**	**T [K]**	**a [Å]**	**c [Å]**	**c/a**	**V [Å^3^]**	**D_cryst._ [nm]**
(**b**) **BTBO:Nd^3+^** **(0.5%)**	27	300.15	8.7529(1)	5.8929(1)	0.67325(1)	390.99(1)	76(1)
	32	305.15	8.7533(1)	5.8929(1)	0.67323(1)	391.02(1)	
	40	313.15	8.7539(1)	5.8930(1)	0.67318(1)	391.08(1)	
	60	333.15	8.7554(1)	5.8931(1)	0.67307(1)	391.22(1)	
	80	353.15	8.7572(1)	5.8930(1)	0.67293(1)	391.38(1)	
	100	373.15	8.7588(1)	5.8932(1)	0.67283(1)	391.53(1)	
	120	393.15	8.7604(1)	5.8932(1)	0.67271(1)	391.68(1)	
	140	413.15	8.7623(1)	5.8934(1)	0.67258(1)	391.86(1)	
	160	433.15	8.7639(1)	5.8934(1)	0.67246(1)	392.01(1)	
	180	453.15	8.7657(1)	5.8935(1)	0.67233(1)	392.17(1)	
	200	473.15	8.7675(1)	5.8935(1)	0.67220(1)	392.34(1)	
	220	493.15	8.7693(1)	5.8937(1)	0.67208(1)	392.51(1)	
**Compound**	**T [°C]**	**T [K]**	**a [Å]**	**c [Å]**	**c/a**	**V [Å^3^]**	**D_cryst._ [nm]**
(**c**) **BTBO:Nd^3+^** **(1.0%)**	27	300.15	8.7520(2)	5.8913(2)	0.67313(2)	390.80(2)	80(1)
	32	305.15	8.7525(2)	5.8912(2)	0.67309(2)	390.84(2)	
	40	313.15	8.7532(2)	5.8914(2)	0.67306(2)	390.92(2)	
	60	333.15	8.7548(2)	5.8913(2)	0.67292(2)	391.04(2)	
	80	353.15	8.7565(2)	5.8915(2)	0.67281(2)	391.21(2)	
	100	373.15	8.7582(2)	5.8916(2)	0.67269(2)	391.37(2)	
	120	393.15	8.7597(2)	5.8916(2)	0.67258(2)	391.51(2)	
	140	413.15	8.7616(2)	5.8917(2)	0.67245(2)	391.69(2)	
	160	433.15	8.7632(2)	5.8918(2)	0.67233(2)	391.84(2)	
	180	453.15	8.7650(2)	5.8918(2)	0.67220(2)	392.00(2)	
	200	473.15	8.7668(2)	5.8919(2)	0.67207(2)	392.17(2)	
	220	493.15	8.7686(2)	5.8920(2)	0.67194(2)	392.33(2)	

**Table 5:** Comparison of thermal expansion anisotropy for selected SFD single crystals with the results of BTBO:Nd^3+^ powders of the current experiment.

Material	Type	TEC [×10^6^/K] (at RT)			Ref.
		α_a_	α_b_	α_c_	
Bi_3_TeBO_9_	polycrystalline	8.53	-	0.24	This work
Bi_2.985_Nd_0.015_TeBO_9_		8.68	-	0.63	
GdAl_3_(BO_3_)_4_	single crystalline	5.30	-	18.8	[90]
La_2_CaB_10_O_19_		8.64	8.39	2.27	[91]
La_2_CaB_10_O_19_:Nd^3+^		8.31	4.13	2.33	[92]
LiNbO_3_		19.2	-	2.7	[93]
YAl_3_(BO_3_)_4_:Yb^3+^		1.4–2.0	-	8.1–9.7	[94]

**Table 6:** Theoretical and experimental atomic contents of Nd and Bi, as well as their Nd/Bi ratio, for the studied Nd^3+^-doped BTBO powders.

Compound	Theor. [at.%]		EDX exp. [at.%]		(Nd/Bi) Theor.	(Nd/Bi) EDX exp.
	Nd	Bi	Nd	Bi		
BTBO:Nd^3+^(0.5%)	0.1071	21.3214	0.03 ± 0.01	13.98 ± 0.38	0.0050	0.0022 ± 0.0006
BTBO:Nd^3+^(1.0%)	0.2143	21.2143	0.12 ± 0.02	12.38 ± 0.33	0.0101	0.0097 ± 0.0015

## Data Availability

Data can be provided upon a reasonable request.
